# Effects of a smartphone-based chatbot intervention on influenza and COVID-19 vaccine uptake among South Asians

**DOI:** 10.1038/s41746-025-02200-1

**Published:** 2025-12-11

**Authors:** Cho Lee Wong, Dorothy Ngo Sheung Chan, Winnie Kwok Wei So, Carmen Wing Han Chan, Kai Chow Choi, Huiyuan Li, Shelby L Garner

**Affiliations:** 1 The Nethersole School of Nursing, Faculty of Medicine, The Chinese University of Hong Kong, Hong Kong, China; 2 School of Nursing, Fudan University, Shanghai, China; 3 Gordon E. Inman College of Nursing, Belmont University, Nashville, TN USA

**Keywords:** Diseases, Health care, Medical research

## Abstract

Smartphone-based chatbot interventions have shown promise in promoting vaccine uptake among South Asian minorities. In this two-arm, cluster-randomised, wait-list controlled trial (Chinese Clinical Trial Registry: ChiCTR2200061503; registration date: June 27^th^, 2022), South Asians (aged 18 or older, from Pakistan, India, or Nepal) who had not received influenza or COVID-19 vaccines in the past year were randomly assigned at the cluster level to intervention or wait-list control groups. The intervention comprised a simple rule-based chatbot integrated into the smartphone application and an on-demand feature allowing users to connect with trained research assistants. Among 610 participants analysed, influenza vaccination rates were significantly higher in the intervention group than the control group immediately after (57.8% vs. 1.3%, p < 0.001) and three months post-intervention (68.0% vs. 2.0%, p < 0.001). Our results suggested that smartphone-based chatbot intervention could be an effective strategy to increase vaccination rates among South Asian ethnic minorities.

## Introduction

Influenza, an acute viral respiratory disease, causes significant morbidity and mortality, particularly among high-risk groups^1^, resulting in a substantial disease burden worldwide^2^. In Hong Kong, there were a total of 942 adult cases of intensive care unit admissions or deaths due to laboratory-confirmed influenza between 1 January 2024 and 1 June 2024, including 607 deaths. Notably, nearly 70% of the severe cases did not receive influenza vaccination^3^. Meanwhile, the COVID-19 pandemic has unprecedentedly impacted the global healthcare system, and Hong Kong is no exception^4^. Given that influenza and COVID-19 share many common characteristics, co-circulating these viruses likely intensifies the burden on hospitalisation and intensive care unit admission^5^. Vaccination has been scientifically proven to be one of the most effective means of preventing severe cases of vaccine-preventable diseases and their complications, such as influenza and COVID-19^6^. It reduces the risks of inpatient admissions and mortality^6^. However, the increasing trend of scepticism toward vaccination, and delays or refusals to receive vaccines has resulted in stagnant immunisation rates, reduced community protection against preventable diseases, and threatened global health sustainability^7^.

Ethnic minorities, defined as groups that differ in race, national origin, religion, or culture from the dominant group in a country, encounter numerous barriers to vaccination for multiple reasons, such as inadequate communication and a lack of trust in governments and health systems^8^. A recent study revealed that less than 50% of major racial/ethnic groups in the US received an influenza vaccination^9^. A systematic review from the UK also indicated that the concerns about the COVID-19 vaccine within ethnic minority communities contribute to low vaccination uptake^10^. In Hong Kong, South Asians, mainly including Indians, Pakistanis, and Nepalis, are one of the most significant (16.5%) and one of the fastest-growing ethnic minority groups^11^. To date, there is a lack of public reporting on vaccination rates among South Asian ethnic minorities. Nevertheless, international data have indicated considerable risks associated with low uptake in these communities.

Vaccine hesitancy presents a significant barrier to achieving the vaccination rates necessary to control the seasonal influenza and COVID-19 pandemic^12^. Characterised by ambivalence or refusal to vaccinate, it was identified by the World Health Organisation (WHO) as one of the top ten global health threats in 2019. Based on the Health Belief Model and Theory of Planned Behaviour, along with the work of the WHO advisory group, researchers have developed the 5 C model (confidence, complacency, constraints, calculation, and collective responsibility) to understand the antecedents of vaccination behaviours^13^. Ethnicity intersects with factors such as low literacy levels and language barriers, which further exacerbate vaccination disparities^14^. Nevertheless, hesitant individuals can often be persuaded of vaccination uptake if provided with accurate information. Therefore, effective interventions that enhance vaccine knowledge and awareness while also addressing access barriers are vital for the success of vaccination programmes targeting ethnic minorities. A recent systematic review involving 23 studies showed that the most common approach to promoting vaccine uptake for ethnic minorities involves educating targeted populations by providing vaccine information^15^. Another systematic review with 13 empirical studies showed that digital technologies (e.g., smartphones) can be a valuable adjunct in improving vaccination rates^16^. Specifically, utilising these technologies to push targeted messages can increase the salience and trustworthiness of the content provided to recipients^16^. However, a text message approach focusing solely on education, without interactive elements, may be the least effective in boosting vaccination coverage. Those not vaccinated early may require different information delivery methods to make informed vaccination decisions^15^. Chatbots, which can be integrated into smartphone-based interventions, may help enhance interactivity and user engagement.

Chatbots are automated conversational agents that simulate human conversation, using text or voice to interact with users in real-time and provide information or support. Simple chatbots are rule-based, relying on pre-written keywords and programmed scripts, which allow providers to disseminate accurate educational information and reminders to users that simulate interactivity experienced by real-life conversations. A recent review indicated that the effectiveness of vaccine chatbots stems from their ability to provide credible and personalised information in real-time, along with the familiarity and accessibility of the chatbot platform. The extent to which interactions feel ‘natural’ to users also contributes to their effectiveness^17^. Smartphone-based chatbots may address barriers faced by ethnic minorities. The multilingual audio and text-to-speech capabilities enhance comprehension for individuals with low literacy^18^. By providing tailored information, chatbots improve relevance and trustworthiness. A recent scoping review of 22 studies indicated that interacting with chatbots can significantly improve COVID-19 vaccination attitudes and notably increase vaccine-related knowledge and intention^19^. Another review, which included six studies in meta-analysis revealed the effectiveness of chatbot interventions to improve vaccination attitudes and intentions^20^. However, evidence on the actual vaccination, optimal dosage and frequency of effective chatbot interventions remains unclear. Moreover, methodological heterogeneity of the included studies exists due to the use of non-experimental study designs, small sample sizes, high attrition of participants, and the lack of an extended follow-up period to assess outcomes^19,20^. Most importantly, no smartphone-based chatbot for promoting influenza and COVID-19 vaccine uptake has been developed and investigated to date for ethnic minority communities. To fill the research gap in this field, we designed a smartphone-based chatbot intervention on the uptake of influenza and COVID-19 vaccines among South Asian ethnic minorities in Hong Kong^18^.

This trial aimed to assess the effects of smartphone-based chatbot intervention on vaccine uptake among South Asian ethnic minorities in Hong Kong. The objectives of this trial were: (1) to assess the effects of smartphone-based chatbot intervention on influenza and COVID-19 vaccine uptake among South Asian ethnic minorities, and (2) to assess the effects of the intervention on the intention to receive influenza and COVID-19 vaccinations, as well as vaccine hesitancy.

## Results

**Fig. 1:**
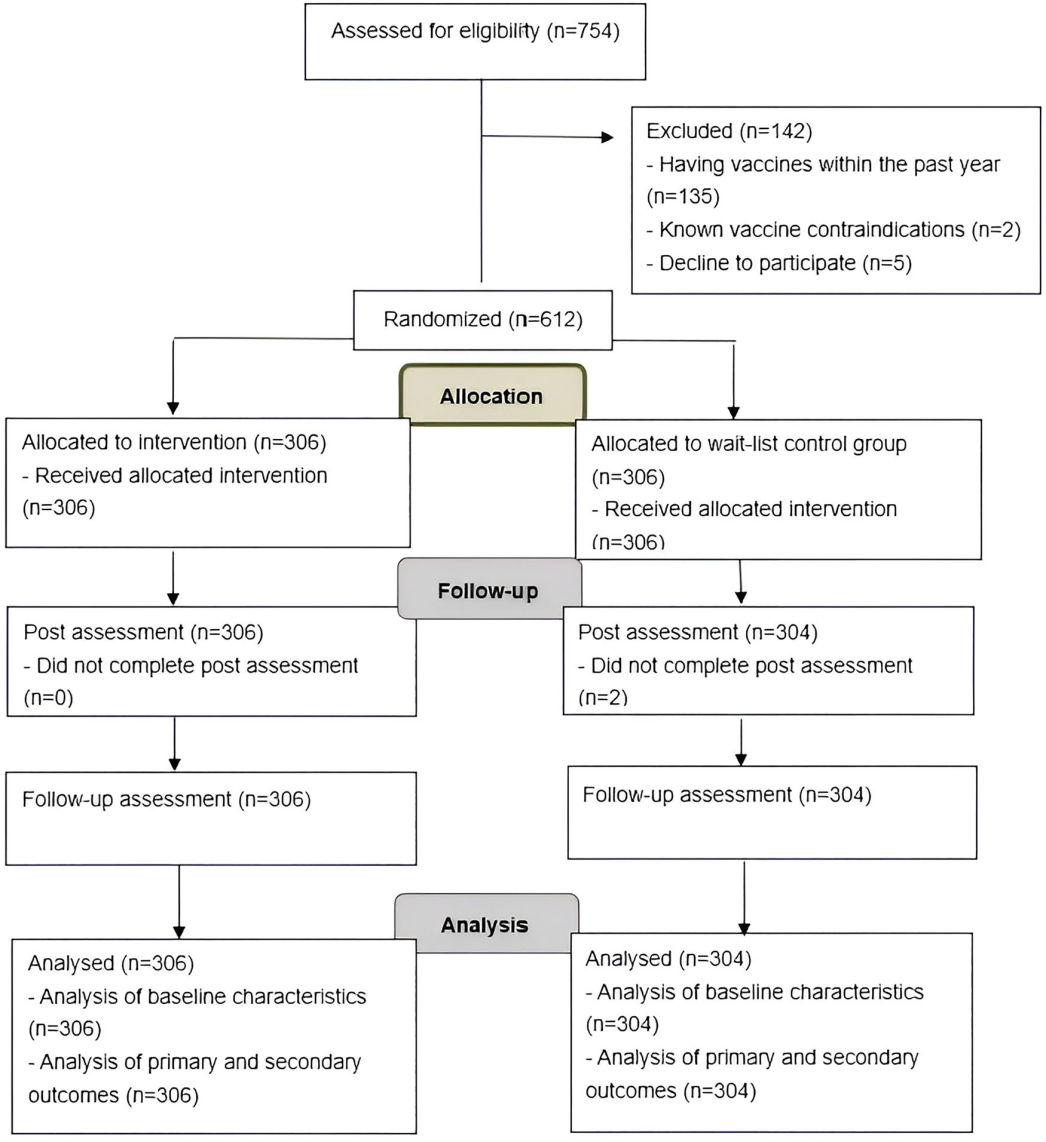
CONSORT flow diagram.

A total of 754 South Asians were approached and screened for eligibility at six non-governmental community centres and ethnic minority associations from May 2023 to March 2024. Of those screened, 142 were excluded: 135 had received influenza and COVID-19 vaccines within the past year, and two had known vaccine contraindications. Among the remaining candidates, five eligible participants declined to participate due to concerns about disclosing their personal information. As a result, 612 participants were successfully recruited and randomly assigned at the cluster level to either the intervention group (n = 306) or the wait-list control group (n = 306), representing a recruitment rate of 81.2%. At T1, two participants from the control group withdrew from the trial because they anticipated leaving Hong Kong for more than three months. Thus, the final analytic sample for primary analysis comprised 610 participants. At three months post-intervention, a total of 610 participants completed the follow-up questionnaire (intervention arm = 306; control arm = 304). The overall retention rate was 99.67% (99.35% for the intervention arm and 100% for the control arm). Regarding participants’ engagement, the mean number of logins was 9.3 (SD = 2.8) times, with a range between 6 and 24 times throughout the intervention period. On average, participants spent approximately 7.9 (SD = 4.3) minutes per session, while session durations varied significantly, ranging from 2.4 minutes to a maximum of 24.6 minutes. In terms of content engagement, participants dedicated the most time to the influenza vaccination programme, averaging about 11.6 minutes per session on this topic. This was followed by information related to COVID-19 vaccines, where participants spent an average of 9.3 minutes per session. For on-demand communication among participants in the intervention group, a total of 18 queries were received from female participants, including three from India, seven from Nepal, and eight from Pakistan. The majority of these inquiries (n = 12) focused on the appropriateness of influenza vaccines for individuals with a history of allergies and chronic illnesses. Additionally, three participants asked about the time interval between doses of the COVID-19 vaccine, while others inquired about vaccine safety during breastfeeding. Figure. 1 shows the CONSORT flow diagram of the trial. The socio-demographic characteristics of the participants were comparable to those of the ethnic minority population in Hong Kong. Details are shown in Table 1, while other baseline characteristics are shown in Table 2. There were no significant differences in any baseline characteristics between the two groups.

**Table 1:** Socio-demographic characteristics of the participants (N = 610)

	Intervention (*n* = 306)	Control (*n* = 304)	*p* value
Age (years)^*^	43.92 (15.10)	41.67 (14.93)	0.06^a^
Sex			0.16^b^
Female	209 (68.3%)	191 (62.8%)	
Male	97 (31.7%)	113 (37.2%)	
Education			0.09^b^
Primary school or below	78 (25.5%)	55 (18.1%)	
Secondary school	104 (34.0%)	119 (39.1%)	
College	69 (22.5%)	63 (20.7%)	
University or above	55 (18.0%)	67 (22.1%)	
Employment status			0.66^b^
Full-time	112 (36.6%)	116 (38.2%)	
Part-time	46 (15.0%)	46 (15.1%)	
Housewife	78 (25.5%)	70 (23.0%)	
Student	23 (7.5%)	32 (10.5%)	
Retired/ unemployed	47 (15.4%)	40 (13.2%)	
Full-Time occupation types			0.25^b^
Elementary occupations	32 (28.6%)	19 (16.4%)	
Service & Sales workers	30 (26.8%)	36 (31.0%)	
Clerical support workers	5 (4.4%)	6 (5.2%)	
Professionals	35 (31.3%)	46 (39.7%)	
Managers & Administrators	10 (8.9%)	9 (7.7%)	
Household income/monthly (HK$)			0.05^b^
< 9999	23 (7.5%)	16 (5.3%)	
10000–19999	56 (18.3%)	74 (24.3%)	
20000–29999	54 (17.6%)	48 (15.8%)	
30000–39999	17 (5.6%)	24 (7.9%)	
>40000	14 (4.6%)	31 (10.2%)	
Do not know	142 (46.4%)	111 (36.5%)	
Marital status			0.07^b^
Single	71 (23.2%)	79 (26.0%)	
Married	214 (69.9%)	216 (71.1%)	
Divorced/Separated/Widowed	21 (6.9%)	9 (2.9%)	
Number of children*	1.34 (1.29)	1.13 (1.48)	0.06^a^
Country of origin			0.60^b^
Pakistan	107 (35.0%)	100 (32.9%)	
India	94 (30.7%)	105 (34.5%)	
Nepal	105 (34.3%)	99 (32.6%)	
Duration stayed in Hong Kong (years)*	19.12 (12.92)	18.12 (11.42)	0.22^a^
Religion			0.08^b^
Hinduism	106 (34.6%)	125 (41.1%)	
Islam	132 (43.2%)	114 (37.5%)	
Buddhism	48 (15.7%)	35 (11.5%)	
Others^c^	20 (6.5%)	30 (9.9%)	
Have you had influenza in the past year?			0.86^b^
Yes	139 (45.4%)	136 (44.7%)	
No/ not sure	167 (54.6%)	168 (55.3%)	
Have you had COVID-19 in the past year?			0.89^b^
Yes	236 (77.1%)	233 (76.6%)	
No/ not sure	70 (22.9%)	71 (23.4%)	
Do you know the Venue of Clinics/Centres providing Influenza/ COVID0-19 vaccination?			0.29^b^
Yes	153 (50.0%)	165 (54.3%)	
No	153 (50.0%)	139 (45.7%)	
First place for seeking health advice when getting sick			0.21^b^
Doctor’s office (General Practitioner)	64 (20.9%)	48 (15.8%)	
Hospital Outpatient Clinics	65 (21.3%)	78 (25.7%)	
Accident & Emergency Department	98 (32.0%)	100 (32.9%)	
Pharmacy Store	29 (9.5%)	18 (5.9%)	
Private clinics using traditional healing methods or herbal medicine	35 (11.4%)	44 (14.5%)	
Others	15 (4.9%)	16 (5.3%)	
Receive recommendations from healthcare professionals about getting influenza/ COVID-19 vaccines			0.11^b^
Yes	74 (24.18%)	91 (29.93%)	
No	232 (75.82%)	213 (70.07%)	
Receive advice from friends about getting Influenza/ COVID-19 vaccines			0.56^b^
Yes	130 (42.48%)	136 (44.74%)	
No	176 (57.52%)	168 (55.26%)	
Receive advice from family about getting Influenza/ COVID-19 vaccines			0.06^b^
Yes	136 (44.44%)	158 (51.97%)	
No	170 (55.56%)	146 (48.03%)	
Receive a reminder letter from medical providers for getting Influenza/ COVID-19			0.19^b^
Yes	49 (14.05%)	61 (20.07%)	
No	257 (85.95%)	243 (79.93%)	
Have you been exposed to influenza/ COVID-19 information through media?			0.07^b^
Yes	118 (38.6%)	139 (45.7%)	
No	188 (61.4%)	165 (54.3%)	

Variables marked with^*^ are presented as mean (standard deviation), otherwise as frequency (%).

^a^Independent t-test

^b^Chi-square test

^c^Sikhism, Christianity or no religion

**Table 2:** Influenza outcomes between the control and intervention groups

		Control	Intervention	Mean difference between groups (95% CI)^b^	p-value
**Primary outcomes**					
***Vaccination uptake***^a^	T1	4 (1.3%)	177 (57.8%)	43.94 (7.57 to 255.01)^c^	**<0**.01*
	T2	6 (2.0%)	208 (68.0%)	34.27 (8.58 to 136.84)^c^	**<0**.01*
**Secondary outcomes**					
***Intention to receive vaccination***	T0	6.16 (3.05)	6.31 (2.71)	NA	NA
	T1	5.71 (2.79)	8.16 (1.80)	2.29 (1.77 to 2.81)	**<0**.**01**
	T2	5.20 (2.77)	8.51 (2.95)	3.15 (1.43 to 4.87)	**<0**.**01**
***Vaccine hesitancy***					
*Confidence*	T0	5.14 (1.46)	5.01 (1.15)	NA	NA
	T1	5.06 (1.37)	5.58 (1.02)	0.66 (−0.09 to 1.40)	0.08
	T2	4.90 (1.39)	6.39 (0.90)	1.62 (0.51 to 2.73)	**<0**.**01**
*Complacency*	T0	3.82 (1.68)	3.64 (1.32)	NA	NA
	T1	3.74 (1.45)	2.77 (1.30)	−0.78 (−2.06 to 0.49)	0.23
	T2	3.64 (1.26)	2.31 (1.87)	−1.15 (−2.78 to 0.48)	0.17
*Constraints*	T0	3.41 (1.96)	3.20 (1.76)	NA	NA
	T1	3.22 (1.62)	2.84 (1.77)	−0.18 (−0.57 to 0.21)	0.37
	T2	3.15 (1.77)	2.36 (1.96)	−0.58 (−2.01 to 0.86)	0.43
*Calculation*	T0	5.47 (1.67)	5.39 (1.22)	NA	NA
	T1	5.46 (1.36)	5.77 (0.95)	0.39 (−0.24 to 1.02)	0.22
	T2	5.50 (1.41)	6.34 (0.95)	0.92 (0.30 to 1.54)	**<0**.**01**
*Collective responsibility*	T0	4.62 (1.27)	4.48 (1.08)	NA	NA
	T1	4.60 (1.13)	4.57 (1.02)	0.11 (−0.30 to 0.52)	0.60
	T2	4.79 (1.20)	4.84 (1.11)	0.19 (−0.31 to 0.69)	0.45

*Remained significant after Holm’s multiplicity adjustment.

^a^Influenza vaccine uptake in two group were presented as number (percentage) and rate ratio (confidence interval).

^b^Mean difference in within-subject change from T0 between groups with adjustment for the baseline values estimated by GEE accounting for the clustering design. *CI* confidence interval. *NA* not applicable.

^c^Rate ratio of vaccination uptake of intervention group relative to control group estimated by GEE accounting for the clustering design.

Table 2 compares the primary and secondary outcomes between participants in the intervention and wait-list control groups. Vaccination rates among participants in the intervention group were significantly higher than those of participants in the control group at T1 (57.8% vs 1.3%, p < 0.01) and T2 (68.0% vs 2.0%, p < 0.01). In addition, significantly greater increase in vaccination intentions were noted in the intervention group than in the control group at both T1 (2.29, 95%CI = 1.77 to 2.81, p < 0.01) and T2 (3.15, 95%CI = 1.43 to 4.87, p < 0.01) with respect to T0. Regarding vaccine hesitancy, all vaccine hesitancy measures showed greater improvements in the intervention group than in the control group at both T1 and T2 relative to T0. In particular, participants in the intervention group reported a significantly greater increase in confidence (1.62, 95%CI = 0.51 to 2.73, p = <0.01) and calculation (0.92, 95%CI = 0.30 to 1.54, p < 0.01) than those in the control group at T2.

A further analysis was conducted to address the potential temporal confounding effect of seasonality of influenza on the influenza vaccination outcome. Enrolment months were categorised into three periods: low season (May to August 2023), high season (September to November 2023), and late season (December 2023 to March 2024). We tested the interaction term between grouping and this recruitment period variable on the outcome of influenza vaccination. This interaction term was not statistically significant at both T1 (p = 0.568) and T2 (p = 0.649). This suggested that the effectiveness of the intervention on influenza vaccination remained consistent across different recruitment seasons and was not overly affected by the time of participant enrolment.

Table 3 compares the primary and secondary outcomes between participants in the intervention and wait-list control groups. Vaccination rates among participants in the intervention group were higher than those of participants in the control group at T1 (2.0% vs 0.3%, p = 0.36) and T2 (2.3% vs 0.7%, p = 0.25). However, no significant differences were noted. A significantly greater increase in vaccination intention was observed in the intervention group than in the control group at T1 (1.32 95%CI = 0.19 to 2.44, p = 0.022), but no significant difference was noted in T2. Regarding vaccine hesitancy, all vaccine hesitancy measures showed more substantial improvements in the intervention group than in the control group at both T1 and T2 concerning T0. In particular, participants in the intervention group reported a significantly more significant increase in confidence (1.61, 95%CI = 0.52 to 2.69, p < 0.01) and calculation (1.02, 95%CI = 0.19 to 1.85, p = 0.016) than those in the control group at T2.

**Table 3:** COVID-19 outcomes between the control and intervention groups

		Control	Intervention	Mean difference between groups (95% CI)^b^	p-value
**Primary outcomes**					
***Vaccination uptake***^a^	T1	1 (0.3%)	6 (2.0%)	4.75 (0.17 to 133.99)^c^	0.36
	T2	2 (0.7%)	7 (2.3%)	3.48 (0.41 to 29.69)^c^	0.25
**Secondary outcomes**					
***Intention to receive vaccination***	T0	6.84 (3.20)	6.81 (2.33)	NA	NA
	T1	6.03 (3.13)	7.32 (2.41)	1.32 (0.19 to 2.44)	**0.02**
	T2	5.19 (3.47)	7.34 (3.47)	2.18 (−0.09 to 4.45)	0.06
***Vaccine hesitancy***					
*Confidence*	T0	5.11 (1.67)	4.89 (1.28)	NA	NA
	T1	5.23 (1.32)	5.60 (1.01)	0.59 (−0.19 to 1.36)	0.14
	T2	4.98 (1.36)	6.38 (0.92)	1.61 (0.52 to 2.69)	**0.01**
*Complacency*	T0	3.87 (1.83)	3.66 (1.27)	NA	NA
	T1	3.60 (1.34)	2.77 (1.31)	−0.62 (−1.88 to 0.63)	0.33
	T2	3.71 (1.39)	2.19 (1.56)	−1.32 (−2.95 to 0.31)	0.11
*Constraints*	T0	3.48 (2.05)	3.24 (1.77)	NA	NA
	T1	3.25 (1.63)	2.86 (1.77)	−0.16 (−0.69 to 0.37)	0.56
	T2	3.15 (1.79)	2.18 (1.84)	−0.73 (−2.34 to 0.88)	0.37
*Calculation*	T0	5.31 (1.80)	5.10 (1.40)	NA	NA
	T1	5.43 (1.33)	5.77 (0.97)	0.55 (−0.26 to 1.36)	0.19
	T2	5.52 (1.36)	6.33 (0.94)	1.02 (0.19 to 1.85)	**0.02**
*Collective responsibility*	T0	4.52 (1.38)	4.35 (1.11)	NA	NA
	T1	4.69 (1.07)	4.56 (1.02)	0.04 (−0.51 to 0.59)	0.90
	T2	4.84 (1.07)	4.84 (1.10)	0.16 (−0.39 to 0.71)	0.56

^a^Influenza vaccine uptake in two group were presented as number (percentage) and rate ratio (confidence interval).

^b^Mean difference in within-subject change from T0 between groups with adjustment for the baseline values estimated by GEE accounting for the clustering design. *CI* confidence interval. *NA* not applicable.

^c^Rate ratio of vaccination uptake of intervention group relative to control group estimated by GEE accounting for the clustering design.

As the subject recruitment and data collection were conducted after all the restrictive measures of COVID-19 were released in Hong Kong, therefore, a sensitivity analysis evaluating the impact of subject recruitment timing before and after easing the restrictions on COVID-19 vaccination uptake was not conducted.

## Discussion

This was the first trial to examine the effects of a smartphone-based chatbot intervention on the uptake of influenza and COVID-19 vaccinations among South Asians in Hong Kong. The results demonstrate that the intervention effectively increased influenza vaccination rates and enhanced participants’ intention to receive both influenza and COVID-19 vaccines. In addition, the intervention significantly reduced vaccine hesitancy, particularly in areas related to vaccine confidence and calculation. These results suggest that smartphone-based chatbot intervention could effectively promote vaccination, especially influenza vaccines, among South Asian ethnic minorities.

Existing chatbot evaluations primarily focused on their acceptability to users, vaccine-related knowledge and attitudes^17,19^. This trial assessed the impact of the chatbot on the behaviour of interest, whether the participant received vaccines or not. Results found that our intervention substantially increased the influenza vaccination uptake among South Asian ethnic minorities, with about 68% of the intervention participants receiving influenza vaccines within three months of the intervention’s completion compared with 2% in the control group. This percentage was comparable to a local chatbot-delivered online intervention targeted at adults 65 years or older, reporting a vaccination uptake rate higher in the intervention group than the control group (50.5% vs 35.5%)^21^. However, their intervention mainly delivered health promotion videos through a messaging application (WhatsApp) based on participants’ stage of change without engaging participants in text or speech communication^21^.

By going beyond simple information delivery, our smartphone-based chatbot intervention offered real-time engagement that effectively addressed evolving concerns about vaccinations and elicited immediate responses, ensuring South Asian participants remained informed and made informed decisions, ^17^ The conversational nature of the chatbot, which makes it easier for participants to relate to the dialogue partner, not only reinforces vaccine knowledge but also fosters a supportive environment that encourages individuals to act to vaccinate^19^.

Moreover, our chatbot assisted participants in finding access to vaccination centres and booking procedures. Behavioural nudges, such as reminders, also create a sense of urgency that motivates individuals to act^22^. These factors likely contribute to higher vaccination rates within South Asian communities, showcasing the potential of targeted digital health interventions for this population. Our chatbot can communicate in common languages (English, Nepali and Urdu) of South Asians, which helped democratise health knowledge and improve vaccine literacy^15,19^. Providing culturally and linguistically tailored information has been essential in overcoming language barriers and enhancing information accessibility for ethnic minority populations^19^.

Interestingly, we found no statistically significant differences between groups regarding COVID-19 vaccine uptake. Notably, there was a low uptake of COVID-19 vaccines with fewer than 10 participants vaccinated during the data collection period from June 2023 to December 2024. This finding raised questions about why our chatbot intervention effectively improved influenza vaccine uptake, while not having a similar impact on COVID-19 vaccination rates. This discrepancy suggested the presence of additional factors influencing COVID-19 vaccination uptake. One potential explanation could be the timing of implementing this intervention. Conducted between 2023 and 2024, this trial coincided with improving the pandemic situations in Hong Kong and easing related restrictions, likely leading to diminished urgency around COVID-19 vaccinations. Furthermore, the revised COVID-19 vaccination guidelines only recommended that individuals in priority groups receive an additional vaccine booster. Indeed, a general decline in COVID-19 vaccination rates was also observed in the population in Hong Kong^23^. Therefore, while our intervention group’s participants reported an increased intention to vaccinate against COVID-19, this did not translate into actual uptake, suggesting that current infection conditions rates and guidelines may influence the decision to receive the vaccine. In contrast, a large-scale preregistered randomised controlled trial in Argentina involving 249,705 participants found that a WhatsApp chatbot designed to simplify the vaccination process – rather than addressing vaccine hesitancy - resulted in more than tripling COVID-19 vaccine uptake compared to the control group, even during a period of low vaccination demand^24^. Therefore, further study is necessary to explore the factors influencing vaccine uptake, particularly during periods of low infection.

Our results indicated that participants in the intervention group expressed a significantly stronger intention to receive influenza and COVID-19 vaccines than those in the control group. This contrasts with a recent systematic review and meta-analysis, which revealed chatbot intervention’s non-significant, trivial effect on vaccination intentions^20^. In that review, the only study demonstrating a significant positive effect on the intent was distinguished for its theoretical approach to intervention design^20^. Similarly, the positive outcomes of our trial in enhancing vaccination intention can likely be attributed to the use of the comprehensive theoretical framework of the 5 C model, which guided our intervention development.

Our smartphone-based chatbot intervention significantly improved both confidence and calculation related to vaccination. By offering tailored information that addresses specific vaccine concerns, chatbots can help alleviate fears and counteract misconceptions, ultimately increasing their confidence in the safety and efficacy of vaccines^25^. This approach is critical in ethnic minorities, where misinformation is often prevalent and where confidence represents a substantial barrier to vaccine uptake^26^. Regarding calculation, the notable effects may stem from our chatbot’s compelling presentation of data and statistics on vaccine effectiveness, and associated risks and benefits. This helped South Asians evaluate how effective the vaccine is likely to be for them, a finding corroborated by another study related to COVID-19^26^.

Unfortunately, our intervention showed limited effectiveness in addressing complacency, constraints, and perceived collective responsibility regarding vaccination. This may be attributed to the chatbot not addressing practical barriers to access, such as transportation, and its inadequate communication of the underlying principles of herd immunity and its contribution to community health benefits^26^. Future research is needed to refine these aspects of the intervention to enhance its effectiveness.

This trial had several limitations. First, only South Asians from India, Nepal and Pakistan were recruited, limiting the generalisability of the results to other ethnic groups. Second, since the study was conducted in Hong Kong, it remains uncertain whether the findings could be applied to the South Asian populations in other countries, warranting further investigation. Third, selection bias might be introduced during the cluster randomisation process due to the limited number of clusters available for randomisation and the potential heterogeneity of participant characteristics in different clusters. Fourth, the relatively small sample size within each cluster precluded subgroup analyses. Additionally, while the current RCT indicates promising effects of smartphone-based intervention on influenza vaccine intention and influenza vaccination within South Asian ethnic minorities, the causal effects of vaccine hesitancy on intention to vaccinate and actual vaccine uptake remain to be further validated. Fifth, the control group received passive usual care without any vaccine-related messaging. Future trials could benefit from including an active control group, such as one receiving standardised text-based information, to better isolate the effects of the chatbot intervention. Sixth, the short follow-up period of three months may not reflect the long-term effect of the intervention. Further studies should be conducted to assess the longer-term impacts of the intervention on vaccination outcomes. In addition, incorporating a cost-effectiveness analysis will provide a more comprehensive understanding of the intervention’s sustainability. Finally, our smartphone-based chatbot is rule-based with predetermined responses. However, recent advances in artificial intelligence-based chatbots have enabled the development of sophisticated chatbots. Therefore, future studies should investigate the additional impact AI-powered chatbots may have.

The target population for this trial included South Asians, who have exhibited low vaccination uptake rates. The results demonstrate that the smartphone-based chatbot intervention effectively promoted vaccination among this population. This innovative approach represents a significant advancement in public health strategies, as it can serve as a valuable tool to increase vaccination rates, particularly among vulnerable and underserved groups. Furthermore, the chatbot’s ability to provide multilingual support ensures that language barriers do not hinder access to essential health information. The intervention improves health equity by making reliable, evidence-based information accessible to those who may otherwise encounter systemic barriers to health information. By leveraging technology in this way, public health campaigns can more effectively engage diverse populations, leading to better health outcomes for ethnic minorities. The smartphone-based chatbot also represents a scalable and effective intervention for promoting vaccination and can be integrated into the healthcare system in future practice.

In conclusion, this is the first study to develop and examine the effectiveness of smartphone-based chatbot intervention on South Asian ethnic minorities. The results demonstrated that the intervention positively influenced vaccine decisions among South Asian ethnic minorities, especially in promoting vaccination uptake and intention and reducing vaccine hesitancy in this population. Future studies can enhance the chatbot’s design and functionality to better address different populations’ unique needs, thereby promoting vaccination more effectively.

## Methods

This project consisted of two phases. Phase I involved the development of a smartphone-based chatbot intervention. Phase II was a cluster-randomised, wait-list controlled trial to implement and evaluate the effects of the intervention.

The trial was conducted in accordance with the Declaration of Helsinki. Ethical approval was secured from the Joint Chinese University of Hong Kong-New Territories East Cluster Clinical Research Ethics Committee (Ref. no.: 2021.688) and registered in the Chinese Clinical Trial Registry (ChiCTR2200061503) on June 27th, 2022. This report follows the Consolidated Standards of Reporting Trials (CONSORT) reporting guideline (Supplementary Note 1).

Eligible participants were provided with comprehensive information about the trial’s details, aims, and objectives, and their right to withdraw at any time. They were assured of anonymity and confidentiality. An information sheet outlining the trial and an informed consent form were provided. Interested participants were asked to sign the consent form and return it to the research assistants, emphasising their voluntary participation and the option to exit the trial without providing a reason.

The smartphone-based chatbot intervention consisted of (1) a simple chatbot built into the smartphone application; and (2) an on-demand option for users to communicate with trained research assistants. The intervention development has been published as a separate study protocol^18^.

An advisory panel was formed, comprising an IT expert, healthcare professionals, educators and South Asian community leaders representing the three ethnic groups: Pakistan, Nepal and India. Three meetings were held to provide feedback on the content of educational modules, questions and pre-specified responses, as well as to ensure that the layout and design of the smartphone application and chatbot were linguistically and culturally relevant to South Asians. “For instance, since the term ‘COVID-19’ is not frequently used in Urdu, the English term ‘COVID-19’ was utilised instead.”

In addition, nine bilingual South Asians—three Indians, three Pakistanis, and three Nepalese—were invited to pilot test the smartphone-based chatbot intervention. The group consisted of five females and four males, aged between 32 and 84. They were selected in consultation with the coordinators of six community centres and associations, ensuring that the sample adequately reflects the target population. As laypersons, they provided feedback on the content, questions, and format, indicating that the language used was appropriate. The participants also suggested incorporating a blue colour scheme and an image of a temple as the background for the smartphone application to represent their culture and community better.

Phase II was a cluster-randomised, wait-list controlled trial. The protocol was registered on the Chinese Clinical Trial Registry (ChiCTR2200061503).

Trained research assistants approached potential subjects at the six participating non-governmental community centres or ethnic minority associations that provide programmes and services for ethnic minorities across different regions in Hong Kong. Following an introductory presentation, South Asians interested in participating in the trial were screened for eligibility.

Inclusion criteria: (1) South Asians (from Pakistan, India or Nepal); (2) aged 18 or older; (3) did not receive influenza or COVID-19 vaccine in the previous year; (4) able to read English, Urdu and Nepali; and (5) possess a smartphone.

Exclusion criteria: (1) participated in any vaccination-related interventions in the previous year; (2) known vaccine contraindications (e.g., a history of severe hypersensitivity to any vaccine component).

The sample size was based on results from a local study that suggested a low influenza vaccine uptake rate (29%) in the general population of Hong Kong^27^. The vaccine uptake rate amongst ethnic minorities could be less than half as compared with the general population^27^. Assuming that our smartphone-based chatbot intervention could increase the uptake rate in ethnic minorities to the level of the general population, 121 participants per each of the intervention and control groups were required to achieve 80% power at a 5% level of significance (two-sided) to detect at least a 15% difference in the uptake rate (30% in the intervention group vs 15% in the control group) between the two groups. To account for the clustering effect by allowing for an intracluster correlation of up to 0.01 and a possible 20% attrition rate, 102 participants should be recruited from each of the six non-governmental community centres or ethnic minority associations.

Six centres and associations agreed to participate in this trial. Each centre or association was randomly assigned to one of the two arms: an intervention group (n = 3) that received a smartphone-based chatbot intervention or a wait-list control group (n = 3) that received the same intervention after the intervention group had completed their session. Participants from each centre/association were assigned to their respective groups to prevent potential contamination among participants. Group allocation was determined by the sequence of enrolment, with unique group identifiers for each centre/association placed in serially numbered sealed opaque envelopes. A statistician implemented a computer-generated randomisation scheme to ensure the concealment of group assignments. Assessments of vaccination uptake and other secondary outcomes were carried out by a research assistant who was blind to the group allocation.

Participants in the control group received the usual care, which includes social services that assist with access to employment and legal support, provide translation and interpretation services, and organise events and activities to foster support networks offered by ethnic minority centres/ associations. They were offered the smartphone-based chatbot intervention immediately after their counterparts had completed the intervention.

**Fig. 2:**
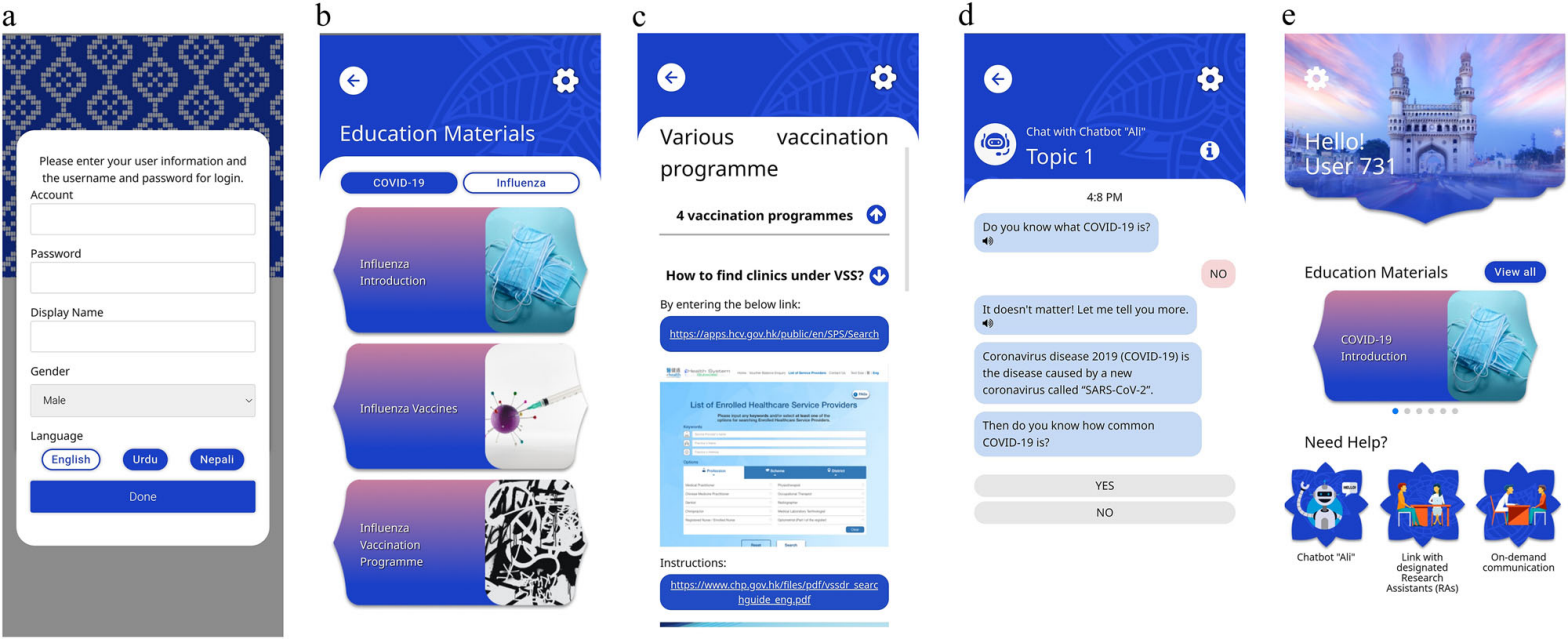
The Screenshot of the chatbot interface. **a** A login page for participants to enter their preferred name and language. **b** Educational topics of Influenza. **c** Content related to various vaccination programme. **d** A conversation with the chatbot. **e** An on-demand option to connect participants with a trained research assistant.

Participants assigned to the intervention group received a smartphone-based chatbot intervention. This intervention was informed by the 5 C model^13^, which connects five constructs to understand the psychological factors influencing vaccine uptake. The primary goal of the intervention was to enhance vaccination uptake by fostering vaccination intention and decreasing hesitancy through five key aspects. A user-friendly chatbot was developed for both Android and IOS platforms to deliver prewritten educational text messages and vaccination reminders. The chatbot set users in-app educational messages and reminders twice a week over four weeks, covering four essential topics. The first topic introduced influenza and COVID-19, presenting their epidemiology and preventive measures aiming to address complacency. The second topic focused on available vaccine options, emphasising effectiveness, safety, necessity and the benefits of vaccination, thereby targeting confidence and collective responsibility. The third topic discussed misconceptions surrounding influenza and COVID-19 vaccines, aimed at overcoming constraints. The final topic provided information about the vaccination programme offered by the Department of Health, and steps for booking a vaccination appointment, aimed at addressing constraints and calculations. The details of the chatbot content mapping to the 5 C model are presented in Supplementary Note 2. To further support users, the chatbot featured an on-demand option that connected participants with trained research assistants in case the chatbot could not satisfactorily address their inquiries. All the content was available in English, Urdu and Nepali, with an optional voice narration feature to facilitate comprehension^18^. Additionally, a ‘members-only’ feature was included to facilitate on-demand communication between project participants and research assistants of the same ethnicity. The screenshot of the chatbot interface is presented in Fig. 2.

Standardisation in the intervention delivery was achieved through a written protocol that research assistants (RA2 and RA3) were required to follow. To enhance their knowledge and competence, they participated in a three-day training session offered by the principal investigator. This training included materials aligned with the latest vaccination guidelines and comprised four modules identical to those in the smartphone application. Additionally, the principal investigator held monthly meetings with the RAs to provide ongoing feedback and support.

Participants were assessed to determine whether they had received influenza and COVID-19 vaccinations, as evidenced by their vaccination records. This method provided an objective evaluation and reduced bias compared to self-report data.

The following secondary outcomes were assessed.

- Intention to receive vaccination: two items asking participants how likely they would take influenza and COVID-19 vaccines when available.
- Vaccine hesitancy: Influenza and COVID-19 vaccine hesitancy were measured by the 5 C Scale separately^17^. The Cronbach’s alpha was 0.83 for influenza and 0.82 for COVID-19, respectively.

Socio-demographic characteristics such as age, ethnic group, and monthly household income were also collected. All the questionnaires were presented in Supplementary Note 3. The reliability, validity, and scoring of all instruments used in this trial can be found in the published protocol^18^.

The RA administered the instruments (except for the socio-demographic questionnaire) blinded to group allocation at three points: baseline (T0) at the trial enrolment, telephone interview post-intervention (T1), and three months post-intervention (T2) through telephone interview. All participants received a HK$200 coupon after completing all assessments.

Data were summarised and presented using appropriate descriptive statistics. The normality of continuous variables was evaluated through skewness and kurtosis statistics, and normal probability plots. The homogeneity of participants’ characteristics between the two groups was assessed by independent t-tests, chi-square tests, or Fisher’s exact tests, which were employed as appropriate.

The effectiveness of the intervention was evaluated by comparing the primary outcome of vaccination uptake rate at T1 and T2 between the two groups. The effects of the intervention on vaccination intention and vaccine hesitancy were explored by comparing the differences in changes in these outcomes at T1 and T2 relative to T0. Generalised estimating equations (GEE) models were employed to estimate the intervention effects on the outcomes with adjustment for correlations owing to the clustering design and repeated measurements^28^. The log binomial and identity link functions were used for the primary uptake outcomes and the secondary continuous outcomes, respectively, in the GEE models. Given the small number of clusters, a small-sample correction for sandwich variance estimate was used in the GEE models^29^. The Holm’s multiplicity adjustment procedure was used to control the family-wise type I error rate owing to multiple comparisons in the primary outcome of vaccination uptake^30^. All the statistical analyses were conducted using SAS release 9.4 (SAS Institute Inc., Cary, NC, USA) with the significance level set at 0.05 (two-sided).

## Supplementary information

Supplementary information

## Data Availability

The datasets used for this study are available from the corresponding authors on reasonable request.

## Code availability

All the statistical analyses were conducted using SAS release 9.4 (SAS Institute Inc., Cary, NC, USA). Codes are not publicly available but may be made available from the corresponding authors on reasonable request.
