# Supplementary material for: Effects of a smartphone-based chatbot intervention on influenza and COVID-19 vaccine uptake among South Asians
Source: NPJ Digit Med. 2025 Dec 11;9:24. doi: 10.1038/s41746-025-02200-1 (PMC12789578; doi:10.1038/s41746-025-02200-1)
Supplement: Supplementary file 1 — Supplementary information [file 41746_2025_2200_MOESM1_ESM.pdf]

Supplementary Information

Supplementary Note 1: CONSORT checklist of information to include when reporting a randomised trial

Supplementary Note 2: The details of the chatbot content mapping to the 5C model and a screenshot of the chatbot interface

Supplementary Note 3: Questionnaires used in the trial

Supplementary Note 1

CONSORT 2010 checklist of information to include when reporting a randomised trial

Section/Topic	Item No	Checklist item	Reported on page No
**Title and abstract**			
	1a	Identification as a randomised trial in the title	1
	1b	Structured summary of trial design, methods, results, and conclusions (for specific guidance see CONSORT for abstracts)	1
**Introduction**			
Background and objectives	2a	Scientific background and explanation of rationale	1
	2b	Specific objectives or hypotheses	2
**Methods**			
Trial design	3a	Description of trial design (such as parallel, factorial) including allocation ratio	9
	3b	Important changes to methods after trial commencement (such as eligibility criteria), with reasons	NA
Participants	4a	Eligibility criteria for participants	10
	4b	Settings and locations where the data were collected	10
Interventions	5	The interventions for each group with sufficient details to allow replication, including how and when they were actually administered	10-11
Outcomes	6a	Completely defined pre-specified primary and secondary outcome measures, including how and when they were assessed	11
	6b	Any changes to trial outcomes after the trial commenced, with reasons	NA
Sample size	7a	How sample size was determined	10
	7b	When applicable, explanation of any interim analyses and stopping guidelines	NA
Randomisation:			
Sequence generation	8a	Method used to generate the random allocation sequence	10
	8b	Type of randomisation; details of any restriction (such as blocking and block size)	10
Allocation concealment mechanism	9	Mechanism used to implement the random allocation sequence (such as sequentially numbered containers), describing any steps taken to conceal the sequence until interventions were	10

		assigned	
Implementation	10	Who generated the random allocation sequence, who enrolled participants, and who assigned participants to interventions	10
Blinding	11a	If done, who was blinded after assignment to interventions (for example, participants, care providers, those assessing outcomes) and how	10
	11b	If relevant, description of the similarity of interventions	NA
Statistical methods	12a	Statistical methods used to compare groups for primary and secondary outcomes	11
	12b	Methods for additional analyses, such as subgroup analyses and adjusted analyses	NA
**Results**			
Participant flow (a diagram is strongly recommended)	13a	For each group, the numbers of participants who were randomly assigned, received intended treatment, and were analysed for the primary outcome	2
	13b	For each group, losses and exclusions after randomisation, together with reasons	2
Recruitment	14a	Dates defining the periods of recruitment and follow-up	2
	14b	Why the trial ended or was stopped	2
Baseline data	15	A table showing baseline demographic and clinical characteristics for each group	4
Numbers analysed	16	For each group, number of participants (denominator) included in each analysis and whether the analysis was by original assigned groups	4
Outcomes and estimation	17a	For each primary and secondary outcome, results for each group, and the estimated effect size and its precision (such as 95% confidence interval)	5-7
	17b	For binary outcomes, presentation of both absolute and relative effect sizes is recommended	NA
Ancillary analyses	18	Results of any other analyses performed, including subgroup analyses and adjusted analyses, distinguishing pre-specified from exploratory	NA
Harms	19	All important harms or unintended effects in each group (for specific guidance see CONSORT for harms)	NA
**Discussion**			
Limitations	20	Trial limitations, addressing sources of potential bias, imprecision, and, if relevant, multiplicity of analyses	8

Generalisability	21	Generalisability (external validity, applicability) of the trial findings	8
Interpretation	22	Interpretation consistent with results, balancing benefits and harms, and considering other relevant evidence	NA
**Other information**			
Registration	23	Registration number and name of trial registry	1, 9
Protocol	24	Where the full trial protocol can be accessed, if available	9
Funding	25	Sources of funding and other support (such as supply of drugs), role of funders	12

*We strongly recommend reading this statement in conjunction with the CONSORT 2010 Explanation and Elaboration for important clarifications on all the items. If relevant, we also recommend reading CONSORT extensions for cluster randomised trials, non-inferiority and equivalence trials, non-pharmacological treatments, herbal interventions, and pragmatic trials. Additional extensions are forthcoming: for those and for up to date references relevant to this checklist, see www.consort-statement.org

Supplementary Note 2: The details of the chatbot content mapping to the 5C model and a screenshot of the chatbot interface

5C Component	Chatbot Content	Description	Screenshot of the smartphone-application interface	Screenshot of dialogue with chatbot
Complacency	Topic 1: Influenza and COVID-19<br>■ What is influenza and COVID-19<br>■ Incidence, mortality, risks, signs and symptoms<br>■ Preventive measures	This content is designed to address complacency by highlighting the risks associated with these diseases, thereby increasing awareness of the need for preventive action.	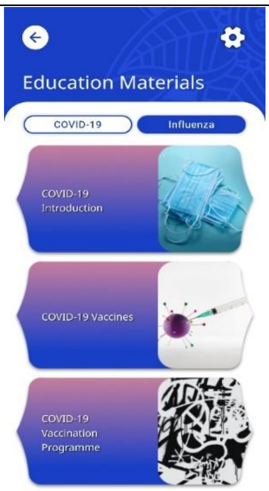	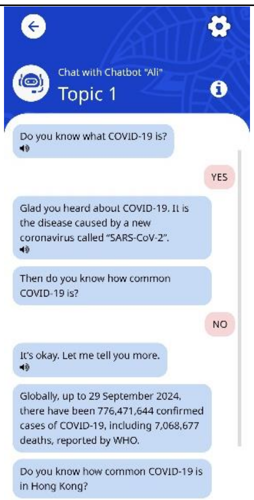
Confidence and collective responsibility	Topic 2: Influenza and COVID-19 vaccines<br>■ Available vaccines options<br>■ Effectiveness, safety, necessity and benefits of vaccination	This directly addresses confidence by providing evidence-based information that reassures users about vaccination. In addition, by framing vaccination as a shared responsibility to protect community health, we aim to foster prosocial motivation and collective	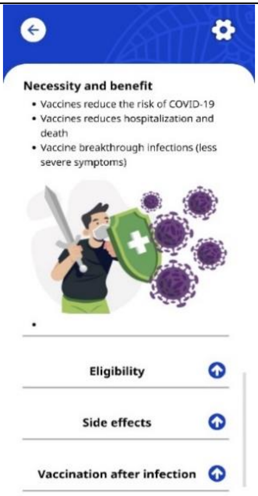	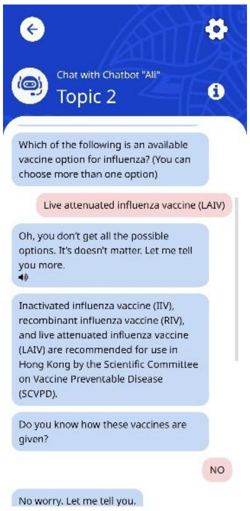

		responsibility among users.		
Constraints	Topic 3: Myths and misconceptions of influenza and COVID-19 vaccines<br>■ Eligibility for vaccination<br>■ Side-effects and contraindications	This helps to overcome barriers (constraints) to vaccination. By addressing misinformation, we aim to empower users to make informed decisions.	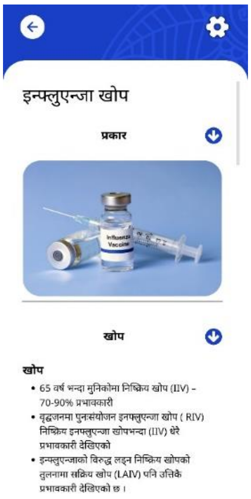<br>Nepali	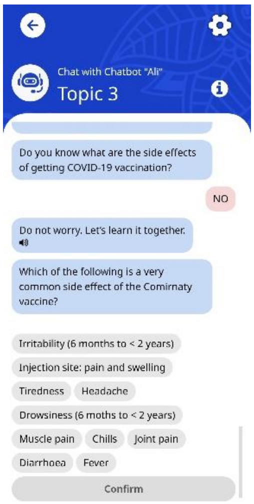
Constraints and calculation	Topic 4: Various vaccination programme<br>■ Contact number and address of specific clinics registered under the influenza and COVID-19 vaccination scheme<br>■ Price and dosages<br>■ Steps to book the vaccination	This practical guidance addresses constraints and calculations and assists users in navigating the logistical aspects of vaccination.	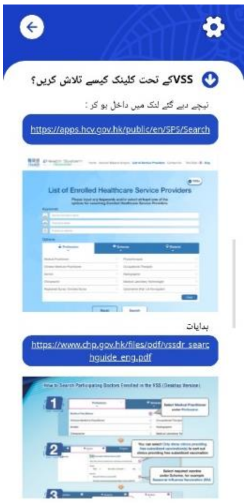<br>Urdu	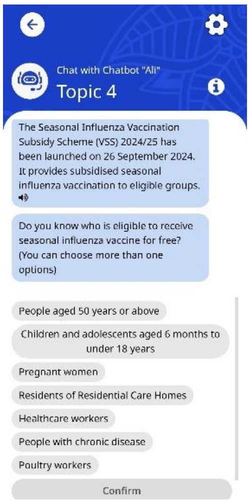

Supplementary Note 3: Questionnaires used in the trial

Intention to receive vaccination

English

1. How likely you will take the influenza vaccine when available?

0 10

(definitely no) (definitely yes)

2. How likely you will take the COVID-19 vaccine when available?

0 10

(definitely no) (definitely yes)

Urdu

1. دستیاب ہونے پر انفلوئنزا ویکسین لینے کا کتنا امکان ہے؟

0 10

یقینی طور پر ن یقینی طور پر ہاں

2. دستیاب ہونے پر COVID-19 ویکسین لینے کا کتنا امکان ہے ؟

0

یقینی طور پر ن یقینی طور پر ہاں

Nepali

1. कती सम्भावना हुन्छ जब तिनीहरूले उपलब्ध हुन्छन् इन्फ्लूएन्जा खोप लिन?

0 10

(पक्कै होईन) (पक्कै हो)

2. उनीहरूले कसरी COVID-19 खोप लिने सम्भावना हुन्छ?

0 10

(पक्कै होईन) (पक्कै हो)

The 5C Scale (English version)

Please evaluate how much you disagree or agree with the following statements." (1 = strongly disagree, 2 = moderately disagree, 3 = slightly disagree, 4 = neutral, 5 = slightly agree, 6 = moderately agree, 7 = strongly agree).

Items	1	2	3	4	5	6	7
**Confidence**							
I am completely confident that vaccines are safe.							
Vaccinations are effective.							
Regarding vaccines, I am confident that public authorities decide in the best interest of the community.							
**Complacency**							
Vaccination is unnecessary because vaccine-preventable diseases are not common anymore.							
My immune system is so strong, it also protects me against diseases.							
Vaccine-preventable diseases are not so severe that I should get vaccinated.							
**Constraints**							
Everyday stress prevents me from getting vaccinated.							
For me, it is inconvenient to receive vaccinations.							
Visiting the doctor's makes me feel uncomfortable; this keeps me from getting vaccinated.							
**Calculation**							
When I think about getting vaccinated, I weigh benefits and risks to make the best decision possible.							
For each and every vaccination, I closely consider whether it is useful for me.							
It is important for me to fully understand the topic of vaccination, before I get vaccinated.							

**Collective responsibility**
When everyone is vaccinated, I don’t have to get vaccinated, too.
I get vaccinated because I can also protect people with a weaker immune system.
Vaccination is a collective action to prevent the spread of diseases.

Socio-demographic characteristics (English version)

To help our research, may I ask you some information about yourself? All information will be treated confidentially and for collective analysis only.

1. What is your date of birth? _______(Date)________(Month)________(Year)

2. What is the highest level of education you completed?

☐ 1. Primary school or below
☐ 2. Secondary school
☐ 3. College
☐ 4. University or above

3. What is your occupation?

☐ 1. Full time,
Please specify: ☐ 1. Managers & administrators
☐ 2. Professionals
☐ 3. Clerical support workers
☐ 4. Service & sales workers
☐ 5. Craft & related workers, plant & machine operators & assemblers
☐ 6. Elementary occupations

☐ 2. Part-time,
Please specify: ☐ 1. Managers & administrators
☐ 2. Professionals
☐ 3. Clerical support workers
☐ 4. Service & sales workers
☐ 5. Craft & related workers, plant & machine operators & assemblers
☐ 6. Elementary occupations

☐ 3. Homemaker/housewife
☐ 4. Student
☐ 5. Freelancer
☐ 6. Unemployed
☐ 7. Retired

4. What is your average monthly household income?

☐ 1. Below $6,000
☐ 2. $6,000 to $9,999
☐ 3. $10,000 to $19,999
☐ 4. $20,000 to $29,999
☐ 5. $30,000 to $39,999
☐ 6. $40,000 to $49,999
☐ 7. $50,000 to $59,999

☐ 8. $60,000 and above
☐ 9. Do not know

5. What is your marital status?
☐ 1. Single
☐ 2. Married
☐ 3. Cohabiting
☐ 4. Divorced/Separated
☐ 5. Widowed

6. Have you given any childbirth before?
☐ 1. Yes, Please state the number of childbirth: ________________
☐ 2. No

7. Please state your country of origin:
☐ 1. Pakistan
☐ 2. India
☐ 3. Nepal

8. Where were you born?
☐ 1. Pakistan
☐ 2. India
☐ 3. Nepal
☐ 4. Hong Kong
☐ 5. Others, please specify: ______________

9. How long have you stayed in Hong Kong? Since ________(year)/ _________ (number of years)

10. What is your religion?
☐ 1. Hinduism
☐ 2. Islam
☐ 3. Sikhism
☐ 4. Buddhism
☐ 5. Christianity
☐ 6. Others, please specify: ______________
☐ 7. No religion

11. Did any of your family members have influenza/ COVID-19 before?
☐ 1. Yes
☐ 2. No
☐ 3. Do not know

12. Have you ever had influenza/ COVID-19 disease before?

☐ 1. Yes, please specify: ______________
☐ 2. No

B. <u>Cues to action</u>

13. Do you know the venue of clinics/centres providing influenza/ COVID-19 vaccination?
☐ 1. Yes, please specify the venue: ____(Go to 13a)
☐ 2. No

13a. Are these clinics easily reached?
☐ 1. Yes
☐ 2. No

14. Where would you seek for health advice when you get sick?
☐ 1. Doctor's office (General Practitioner)
☐ 2. Hospital Outpatient Clinics
☐ 3. Accident and Emergency Department
☐ 4. Pharmacy store
☐ 5. Private clinic using traditional healing methods or herbal medicines
☐ 6. Others, please specify: ____

15. Do you have regular body check-up?
☐ 1. Yes
☐ 2. No

16. Do you have any health insurance?
☐ 1. Yes
☐ 2. No

17. Does any doctor suggest you to have influenza/ COVID-19 vaccination?
☐ 1. Yes
☐ 2. No

18. Do your friends suggest you to have influenza/ COVID-19 vaccination?
☐ 1. Yes
☐ 2. No

19. Does your family suggest you to have influenza/ COVID-19 vaccination?
☐ 1. Yes
☐ 2. No

20. Have you ever received a reminder letter from doctor or healthcare organization for influenza/ COVID-19 vaccination?
☐ 1. Yes

☐ 2. No

21. Have you ever exposed to influenza/ COVID-19 information through media (e.g. TV advertisement, pamphlet, seminar)?

☐ 1. Yes, please specify the source of information: ____

☐ 2. No
